# The instantly blocking-based fluorescent immunochromatographic assay for the detection of SARS-CoV-2 neutralizing antibody

**DOI:** 10.3389/fcimb.2023.1203625

**Published:** 2023-09-05

**Authors:** Yizhe Li, Jinyong He, Ying Zhang, Dan Liang, Jiaqi Zhang, Ruili Ji, Yue Wu, Zejie Su, Changwen Ke, Ning Xu, Yong Tang, Jianhua Xu

**Affiliations:** ^1^ Department of Laboratory Medicine, Shunde Hospital of Guangzhou University of Chinese Medicine, Foshan, Guangdong, China; ^2^ Department of Bioengineering, Guangdong Province Engineering Research Center of Antibody Drug and Immunoassay, Jinan University, Guangzhou, Guangdong, China; ^3^ Guangdong Provincial Institute of Public Health, Guangdong Center for Disease Control and Prevention, Guangzhou, Guangdong, China; ^4^ Department of Laboratory Science, The Second Affiliated Hospital of Guangzhou University of Chinese Medicine, Guangzhou, Guangdong, China; ^5^ Maoming Hospital of Guangzhou University of Chinese Medicine, Maoming, Guangdong, China

**Keywords:** COVID-19 vaccine, conventional virus neutralization test, neutralizing antibody, fluorescent lateral flow immunochromatographic assay, receptor binding domain

## Abstract

**Introduction:**

At present, there is an urgent need for the rapid and accurate detection of severe acute respiratory syndrome coronavirus 2 (SARS-CoV-2) neutralizing antibodies (NAbs) to evaluate the ability of the human body to resist coronavirus disease 2019 (COVID-19) after infection or vaccination. The current gold standard for neutralizing antibody detection is the conventional virus neutralization test (cVNT), which requires live pathogens and biosafety level-3 (BSL-3) laboratories, making it difficult for this method to meet the requirements of large-scale routine detection. Therefore, this study established a time-resolved fluorescence-blocking lateral flow immunochromatographic assay (TRF-BLFIA) that enables accurate, rapid quantification of NAbs in subjects.

**Methods:**

This assay utilizes the characteristic that SARS-CoV-2 neutralizing antibody can specifically block the binding of the receptor-binding domain (RBD) of the SARS-CoV-2 spike protein and angiotensin-converting enzyme 2 (ACE2) to rapidly detect the content of neutralizing antibody in COVID-19-infected patients and vaccine recipients.

**Results:**

When 356 samples of vaccine recipients were measured, the coincidence rate between this method and cVNT was 88.76%, which was higher than the coincidence rate of 76.97% between cVNT and a conventional chemiluminescence immunoassay detecting overall binding anti-Spike-IgG. More importantly, this assay does not need to be carried out in BSL-2 or 3 laboratories.

**Discussion:**

Therefore, this product can detect NAbs in COVID-19 patients and provide a reference for the prognosis and outcome of patients. Simultaneously, it can also be applied to large-scale detection to better meet the needs of neutralizing antibody detection after vaccination, making it an effective tool to evaluate the immunoprotective effect of COVID-19 vaccines.

## 1 Introduction

Since coronavirus disease 2019 (COVID-19) was first reported in December 2019, it has had an unprecedented devastating impact on global society and its economy. COVID-19 is a new type of infectious disease caused by severe acute respiratory syndrome coronavirus 2 (SARS-CoV-2) (Sun et al., 2020; Gavriatopoulou et al., 2021). Its clinical symptoms mainly include fever, cough, and dyspnea (Juan et al., 2020). Moreover, it can lead to acute respiratory distress syndrome and, in worst case scenarios, to organ failure or death (Madjunkov et al., 2020). Severe acute respiratory syndrome coronavirus 2 (SARS-CoV-2) is an enveloped single-stranded positive-sense RNA virus with four main structural proteins: S protein (spike protein), N protein (nucleocapsid protein), E protein (envelope protein), and M protein (membrane protein) (Freeman and Swartz, 2020; Lu S. et al., 2021). Among them, the S protein is key for the invasion of host cells and is a trimeric transmembrane glycoprotein composed of S1/S2 heterodimers. It can recognize the host cell receptor angiotensin converting enzyme 2 (ACE2) and mediate fusion with the cell membrane. A C-terminal receptor binding domain (RBD) of the SARS-CoV-2 spike protein is directly involved in the recognition of the host receptor and mediates the invasion of the virus into the host cell (Ge et al., 2021). The human immune system produces corresponding antibodies after stimulation by SARS-CoV-2. Neutralizing antibodies (NAbs) are specific antibodies that act against SARS-CoV-2 neutralizing epitopes. They can directly target the RBD of the SARS-CoV-2 spike protein epitope and prevent the binding of SARS-CoV-2 RBD to its host cell receptor ACE2, thereby protecting the human body (Ou et al., 2020; Shi et al., 2020). Therefore, there are SARS-CoV-2-specific neutralizing antibodies in the blood of COVID-19 convalescent patients and vaccinated patients (Duan et al., 2020; Wu et al., 2021). In addition, some articles note that neutralizing antibodies have the potential to block the virus from infecting target cells, and monoclonal antibodies have a clear mechanism of action and are easy to prepare on a large scale, which is the focus of research on SARS-CoV-2 therapeutic drugs (Barnes et al., 2020; Wan et al., 2020). Therefore, a rapid and accurate test for SARS-CoV-2 neutralizing antibodies is particularly important. By testing neutralizing antibodies, we cannot only evaluate the immune protection after COVID-19 infection and vaccination and the requirement for a booster vaccination but also quickly detect the transferred neutralizing monoclonal antibodies as part of COVID-19 treatment. This type of testing can better meet the needs of the effectiveness evaluation of neutralizing antibodies brought by the large-scale diagnosis and treatment of COVID-19 and vaccination.

The current laboratory gold standard for SARS-CoV-2 neutralization antibody detection is the conventional virus neutralization test (cVNT), which uses a sample of quantitative live virus mixed with the same amount of serum of different dilutions to plaque reduction neutralization test (PRNT), which analyzes the level of neutralizing antibody content in serum samples by detecting cytopathic effect (CPE) (Chen CZ. et al., 2020; Perera et al., 2020; Tan et al., 2020; Zhu et al., 2020; Valcourt et al., 2021). Although this method has very good sensitivity and specificity, the cVNT method requires active novel coronavirus culture and identification, which must be performed by professionals in the biosafety level-3 laboratory (BSL-3). Each test takes 4–7 days, the BSL-3 resources in China are limited, and the method is a tedious manual operation, which is inefficient and cannot be carried out in conventional laboratories. These shortcomings greatly limit its large-scale use (Tan et al., 2020). To improve the safety of the experiment and reduce the requirements for the experimental environment, pseudovirus-based VNT (pVNT) was developed for neutralizing antibody detection. pVNT can be a better alternative to cVNT and only needs to be performed in BSL-2, but it still requires the use of live viruses and cells (Tan et al., 2020; Liu K-T. et al., 2022). A surrogate VNT (sVNT) can also detect NAbs without the need for any live virus or cells, but it can be completed within 1–2 h in a BSL-1 laboratory(Bošnjak B, Stein SC, Willenzon S, et al. Low serum neutralizing anti-SARS-CoV-2 S antibody levels in mildly affected COVID-19 convalescent patients revealed by two different detection methods. Cell Mol Immunol. 2021;18 (Madjunkov et al., 2020):936-944. doi:10.1038/s41423-020-00573-9), which is not suitable for large-scale testing at the grass-roots level (Tan et al., 2020), including enzyme-linked immunosorbent assay (ELISA) and microparticle chemiluminescence immunoassay (MCLIA) (Peterhoff et al., 2021; Zhang et al., 2021a). Although neutralizing antibody ELISA techniques have developed high sensitivity and good repeatability (Tan et al., 2020; Bošnjak et al., 2021; Behrens et al., 2022), they have the disadvantages of long reaction time (You et al., 2021). The MCLIA is an expensive and complex manual operation, which limits the wide application of SARS-CoV-2 neutralization antibody detection (Elledge et al., 2021). In recent years, lateral flow immunochromatography (LFIC) has been widely used in real-time detection in related industries because of its low cost, simple operation, and rapid detection (Jiang et al., 2019; Urusov et al., 2019; Chen H. et al., 2020). Given the low level of neutralizing antibodies in the early stages of 2019-nCoV infection, more sensitive fluorescent immunochromatographic detection techniques need to be developed to better monitor the dynamic range of neutralizing antibodies in the COVID-19 vaccine audience (Suryoprabowo et al., 2021). Polystyrene nanoparticles labeled with europium nanoparticles (EuNPs) are a new type of fluorescent signal based on the marking of elements of the niobium system. This signal has unique fluorescence characteristics, such as a narrow emission peak, high quantum yield, large Stokes shift, long fluorescence lifetime, and low environmental interference (Chen et al., 2021; Liu M. et al., 2022). Time-resolved fluorescence immunoassay technology with EuNPs as signals is widely used in clinical medical diagnosis, environmental analysis, and food monitoring due to its fast, sensitive, economical, and portable characteristics (Sun et al., 2018; Lu J. et al., 2021).

In this study, we used EuNPs labeled with the RBD of the SARS-CoV-2 spike protein as a fluorescent probe to develop a TRF-BLFIA for the rapid detection of body neutralizing antibody content in COVID-19-infected and vaccine recipients. The cutoff value of the newly established method was determined using relevant clinical samples, and the coincidence rates of negative and positive results of the two methods were compared. The neutralizing antibody titers (NAb titers) of these clinical samples were determined by cVNT by Guangdong Provincial Centers for Disease Control and Prevention. When 356 serum samples from vaccine recipients were detected, the total coincidence rate between this method and cVNT was 88.76%. In addition, it has been reported that the SARS-CoV-2 spike-specific immunoglobulin G (S-IgG) antibody response after immunization might be an alternative biomarker for assessing COVID-19 vaccine efficacy (Racine and Winslow, 2009; Murin et al., 2019; Banga Ndzouboukou et al., 2021). Thus, we detected the level of S-IgG in these clinical samples, and the total coincidence rate with cVNT was only 76.97%. TRF-BLFIA can better detect the content of COVID-19 neutralizing antibodies in serum samples than a conventional chemiluminescence immunoassay detecting overall binding anti-Spike-IgG, and it is a practical tool to evaluate the prognosis of COVID-19 patients and the immune efficacy of the COVID-19 vaccine.

## 2 Materials and methods

### 2.1 Materials and chemicals

Europium chelate fluorescent microspheres (particle size, 312 nm; excitation wavelength, 365 nm; emission wavelength, 610 nm), 1-(3-dimethylaminopropyl)-3-ethylcarbodiimide hydrochloride (EDC),s and N-hydroxysulfosuccinimide sodium salt (NHS) were purchased from Thermo Fisher Scientific, USA. Nitrocellulose (NC) membranes (CN140), conjugation pads, sample pads, soleplates, and absorbent paper were purchased from Millipore (Shanghai, China). Goat anti-chicken IgY and chicken IgY antibodies were obtained from Hangzhou Kitgen Biotechnology Co., Ltd., China. The RBD-Ag3, anti-CoV-19 IgG, and ACE2-RP2 proteins were obtained from Fapon Biological Co., Ltd. Carboxyl-functionalized europium chelate nanoparticles (EuNPs) (Catalogue Number 93470720011150) were obtained from Guangzhou Ewell Biotechnology Co. Ltd. All ultrapure water used in this study was obtained from the Milli-Q ultrapure system (Millipore, MA, USA). All chemicals used were pure or higher for analysis.

### 2.2 Apparatus

A field-emission transmission electron microscope (TEM, Philips, Holland) and centrifuge (Beckman, Germany) were used. An XYZ 3200 series dispense system (Bio-Dot Scientific Equipment, Pvt. Ltd., USA) and a programmable HGS201 strip cutter (purchased locally in Shanghai, China) were also used in this study. A fluorescence ICS reader (AFS330 M, Guangzhou Lab Biotech, China) was used to record the fluorescence signal of TRF-BLFIA.

### 2.3 Preparation of EuNPs-RBD-Ag3 and EuNPs-goat anti-chicken IgY

The coupling process of EuNPs-RBD-Ag3 was as follows: 10 µL EuNPs was added to 990 µL 2-Morpholinoethanesulphonic acid (MES) buffer (0.1 M, pH 6.0). Then, 10 µL of 10 mg mL^−1^ EDC and 50 mg mL^−1^ NHS were added to activate the microspheres, and the entire reaction system was rotated and reacted for 30 min in the dark and then centrifuged at 15,000 rpm for 30 min to remove the supernatant. Next, 1 mL MES was added to suspend the precipitate, and 15 µg RBD-Ag3 and 25 µg goat anti-chicken IgY were added to label the fluorescent microspheres. After rotating for 60 min, 10 µL of 10% bovine albumin (BSA) was added to seal the surface of the microspheres, rotated to mix for 30 min, and centrifuged at 15,000 rpm for 30 min. After centrifugation, the labeled preservation solution containing BSA, sucrose, trehalose, and sodium casein was used to resuspend the labeled substance by ultrasound and then stored at 4°C for later use.

### 2.4 Preparation of the test line and control line

ACE2-RP2 and chicken IgY were diluted with phosphate buffered saline (PBS) containing trehalose, and then both were coated on the NC membrane with a coating amount of 1 µg cm^−1^ to form a test line and a quality control line, with an interval of 5 mm. Then, the coated NC membrane was placed in a 37°C oven to dry for more than 12 h.

### 2.5 Fabrication of the TRF-BLFIA for SARS-CoV-2 neutralizing antibody detection

The SARS-CoV-2 neutralizing antibody test strip is composed of a sample pad, binding pad, NC membrane, absorbent paper, and plastic bottom plate. A certain labeling amount of EuNPs-RBD-Ag3 and EuNPs-goat anti-chicken IgY was sprayed on the binding pad by a gold-spraying scribing machine and placed in a 37°C oven to dry. Then, ACE2-RP2 and chicken IgY were quantitatively distributed to specific areas of the NC membrane as the detection line and quality control line and dried overnight at 37°C for later use. All parts are pasted on the plastic bottom plate in the order of sample pad, binding pad, NC membrane, and absorbent paper. Each part overlaps approximately 2 mm to ensure that the sample can smoothly complete the entire flow process. Finally, the HGS201 programmable strip cutter cuts the test strips into 4-mm wide test strips and places them in a plastic cartridge for sample determination.

### 2.6 Fluorescence immunoassay analysis system

The laboratory cooperated with Guangzhou Labsim Biotechnology Co., Ltd. to develop a dry immunofluorescence analysis system to quantify the fluorescence value of each test line and control line. The dry immunofluorescence analysis instrument was composed of excitation light and a receiver. When the fluorescence test strip is inserted into the dry immunofluorescence analyzer, the excitation light will excite the passing detection line and the quality control line to emit 615 nm fluorescence, and the acceptor will receive the intensity of this fluorescence and transfer the received fluorescence intensity to the computer. The corresponding software in the computer combines the built-in neutralizing antibody standard curve to generate the corresponding neutralizing antibody concentration value of the tested sample.

### 2.7 Microneutralization assay

Fourfold serial dilutions (from 1:4 to 1:1,024) of heat-inactivated serum were mixed 1:1 with a SARS-CoV-2 suspension of 100 TCID_50_ per mL and preincubated for 120 min at 37°C in a 5% CO_2_ incubator. Then, 100 µL of the mixture at each dilution was added to a cell plate containing a semiconfluent Vero E6 monolayer. The plates were incubated for 4 days at 37°C in a humidified atmosphere with 5% CO_2_. After 4 days of incubation, the plates were washed and inspected with an inverted optical microscope. The highest serum dilution that protected more than 50% of the cells from the cytopathic effect (CPE) was taken as the neutralization titer. The highest dilution protects more than half of the cells in the CPE as NAb titers.

### 2.8 Data collection of SARS-CoV-2 S-IgG levels

Levels of SARS-CoV-2 S-IgG were assessed by using the novel coronavirus (2019-nCoV) IgG antibody diagnostic kit and the AutoLumo A2000 Plus automatic chemiluminescence analyzer (Autobio Diagnostics, Zhengzhou, China) according to the manufacturer’s instructions. The S-IgG levels are presented as chemiluminescence values divided by the cutoff (S/CO). The manufacturer’s threshold for positivity is set to S/CO value ≥ 1.0 levels.

### 2.9 Detection method of clinical samples of the TRF-BLFIA for SARS-CoV-2 neutralizing antibody

The serum of the COVID-19 vaccinees was centrifuged, and then, 20 µL of the supernatant was taken, added to 80 µL of sample diluent (PBS containing 0.5% Tween20), and shaken to mix. The 100-µL diluted sample is added to the test strip sample pad, and the labeling compound on the binding pad will migrate to the absorbent paper due to capillary action and pass through the detection line ACE2-RP2 and the quality control line chicken IgY. After 20 min, the test paper card was inserted into the fluorescence ICS card reader to detect T- and C-line fluorescent signals. Substituting the ratio of the fluorescence signal intensities at the T and C lines (F_T_/F_C_) into the calibration curve can obtain the negative and positive levels of the COVID-19 neutralizing antibodies in each sample.

### 2.10 Performance of the TRF-BLFIA for SARS-CoV-2 neutralizing antibody detection

#### 2.10.1 The calibration curve of TRF-BLFIA

Fluorescence immunochromatographic strips were tested using anti-CoV-19 IgG standards at concentrations of 0 ng mL^−1^, 7.8 ng mL^−1^, 15.6 ng mL^−1^, 31.3 ng mL^−1^, 62.5 ng mL^−1^, 125 ng mL^−1^, 250 ng mL^−1^, 500 ng mL^−1^, 1,000 ng mL^−1^, 2,000 ng mL^−1^, 5,000 ng mL^−1^, 10,000 ng mL^−1^, or 20,000 ng mL^−1^. Each concentration was repeated three times, the logarithm of the concentration of anti-CoV-19 IgG was used as the abscissa, and the logarithm of the corresponding T/C mean was used as the ordinate to draw a standard curve.

#### 2.10.2 Determination of TRF-BLFIA specificity

TRF-BLFIA was tested for specificity using pathogens from Staphylococcus aureus and Staphylococcus haemolyticus, and Escherichia coli, Enterococcus faecium, Candida albicans, Klebsiella pneumoniae, Stenotrophomonas maltophilia, and Aeromonas hydrophila and compared with the negative control serum and the positive quality control serum. The results were detected using a fluorescence strip reader after 20 min.

#### 2.10.3 Determination of TRF-BLFIA accuracy

The control substance was diluted to 62.5 ng mL^−1^, 250 ng mL^−1^, 500 ng mL^−1^, 1,000 ng mL^−1^, and 5,000 ng mL^−1^ and detected by a TRF-BLFIA strip. Each concentration was determined repeatedly five times. The mean (M), standard deviation (SD), and the rate of recovery of each concentration were calculated, and the accuracy of the analysis was obtained.

#### 2.10.4 Determination of TRF-BLFIA repeatability

Samples with concentrations of 250 ng mL^−1^, 500 ng mL^−1^, and 1,000 ng mL^−1^ were added to the same batch of test strips in turn, and each sample was repeated five times. Samples with concentrations of 250 ng mL^−1^, 500 ng mL^−1^, and 1,000 ng mL^−1^ were added, and five batches of test strips were repeated. The mean (M), standard deviation (SD), and coefficient of variation (CV) were calculated, and the intra- and interbatch repeatability of the test strips was evaluated.

#### 2.10.5 Determination of TRF-BLFIA stability

The prepared fluorescent immunochromatographic test strips were placed at 25°C and 50°C for 0, 1, 4, 7, 14, 23, 28, 42, 23, 28, 42, 56, 70, and 91 days, and PBS containing 0.5% Tween20 was detected. The concentrations of 500 ng mL^−1^ and 2,000 ng mL^−1^ quality control material were repeated three times to investigate the accelerated stability of the immunofluorescence detection method.

### 2.11 Clinical sample analysis of the TRF-BLFIA

This study was approved by the Medical Ethics Committee of Shunde Hospital of Guangzhou University of Chinese Medicine (ethics approval number: KY-2020128). Serum samples from multiple COVID-19 vaccination patients at different time points were obtained, and each serum sample was stored at −80°C for later use. The cVNT of each serum sample was completed by the Guangdong Provincial Centers for Disease Control and Prevention to independently detect the content of neutralizing antibodies in each serum sample. Fluorescence immunochromatography was used to detect 356 serum samples, including 216 negative samples and 140 positive samples, which had been tested by cVNT. The consistency between this method and cVNT was analyzed by SPSS 26.0 software. The cutoff value of the TRF-BLFIA was determined according to the receiver operating characteristic curve (ROC curve), and the positive coincidence rate, negative coincidence rate, and Kappa coefficient were calculated.

### 2.12 Data analysis

All data were analyzed by SPSS 26.0 software, and the corresponding charts were generated by Origin Lab 9.0 and GraphPad Prism 9.0.

## 3 Results and discussion

### 3.1 Principle of the TRF-BLFIA

**Figure 1:**
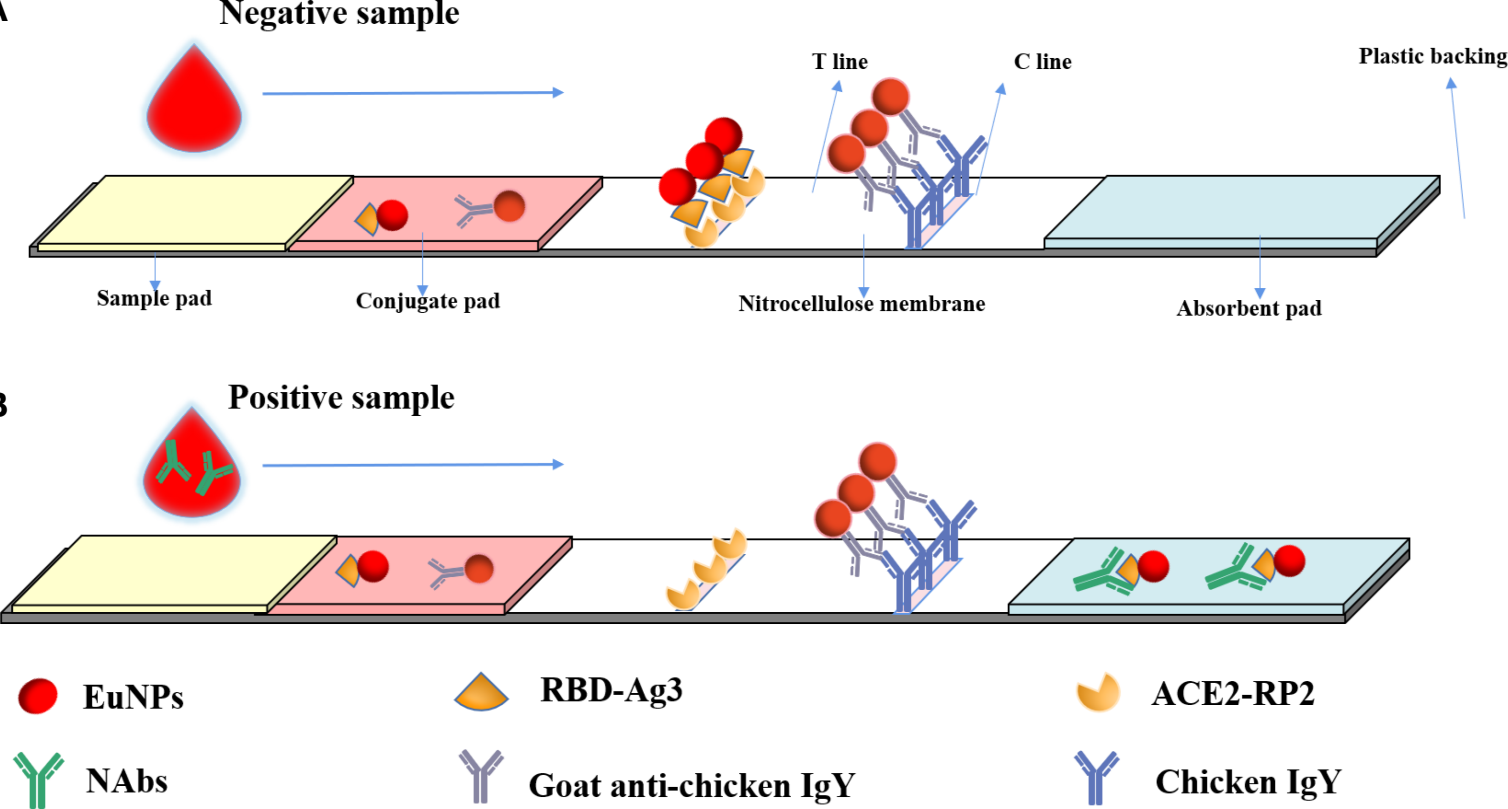
Principal diagram of the fluorescence immunochromatographic assay for the detection of neutralizing antibodies against SARS CoV-2 by the blocking method. **(A)** When a negative reaction was added to the sample pad, the fluorescence signal was observed on both the T line and C line. **(B)** In a positive sample, the fluorescence signal on the T line would be low or absent, but a C-line signal would be observed.

After humans are infected with SARS-CoV-2 or injected with a SARS-CoV-2-inactivated virus vaccine, the immune system produces specific neutralizing antibodies against SARS-CoV-2 neutralizing epitopes after stimulation by related proteins. These specific antibodies can directly target the neutralizing epitopes of the virus, making the virus lose its ability to bind to ACE2 receptors. When the researchers tested blood samples, they precipitated blood cells and other related substances in the blood by centrifugation, and SARS-CoV-2 neutralizing antibodies were distributed in the serum at the upper blood level. As shown in **Figure 1**, the processed sample pad, binding pad, NC membrane, and absorbent paper are combined to form a neutralizing antibody detection test strip. The binding pad was sprayed with a fixed amount of EuNP-RBD and EuNP-goat anti-chicken IgY mixed markers. The NC membrane was sequentially coated with a test line (T line) composed of ACE2 and a quality control line (C line) composed of chicken IgY. When the diluted serum sample is added to the sample pad, if the tested serum sample does not contain or contains a lower concentration of SARS-CoV-2 neutralizing antibody, most of the corresponding EuNPs-RBD on the binding pad will be bound by the ACE2 protein on the T line. If the serum sample contains a higher concentration of COVID-19 neutralizing antibody, most of the EuNP-RBD markers on the binding pad will not bind to ACE2 on the T line due to the binding of the neutralizing antibody in the serum sample. The higher the concentration of the neutralizing antibody of COVID-19 in the serum sample, the less the corresponding EuNPs-RBD will bind to the corresponding ACE2 on the T line, resulting in a weaker fluorescence signal (**Figure 1**). However, no matter how the concentration of COVID-19 neutralizing antibody in the serum changes, the fluorescence signal of C line will basically remain unchanged, and the ratio of the fluorescence signal of the corresponding T line to the fluorescence signal of C line will be inversely proportional to the concentration of COVID-19 neutralizing antibody in the serum sample.

### 3.2 Characterization of the TRF-BLFIA

**Figure 2:**
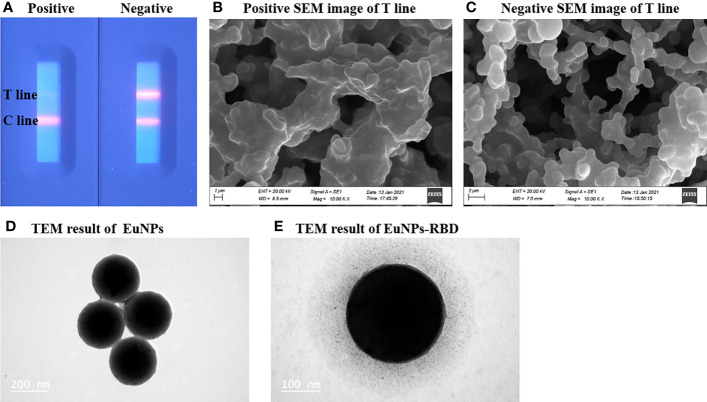
Characterization of EuNPs and EuNP-RBD. **(A)** Positive and negative assay fluorescence photographs. **(B)** Positive assay SEM image of the T line. **(C)** Negative assay SEM image of the T line. **(D)** TEM result of EuNPs. **(E)** TEM result of EuNP-RBD.

The successful preparation of the EuNP-RBD fluorescent probe is the key to this detection system. To confirm the selectivity of TRF-BLFIA to the COVID-19 vaccination, the team used the negative serum samples and the positive serum samples of the COVID-19 vaccine recipients and added them to the test strips prepared by this method. After 20 min, the results were recorded using a fluorescent ICS reader. As shown in **Figure 2**, there is a strong fluorescent signal on the T-line in the case of drips of unvaccinated serum, but the signal disappears in the test strip of the positive serum sample of the COVID-19 vaccine recipients (**Figure 2A**). To determine whether the combination of EuNP-RBD and ACE2 actually occurred on the detection line, the research group used scanning electron microscopy to obtain SEM images of the inspection line area of the two samples. In the case of dripping the negative serum samples, the research group observed that the corresponding detection line area formed an immune compound (**Figure 2B**), and for the strong positive serum samples of the SARS-CoV-2 vaccine recipients, almost no immune complexes were observed on the NC membrane (**Figure 2C**). The combination of EuNPs and RBD was determined by transmission electron microscopy. **Figure 2D** shows unlabeled EuNPs. **Figure 2E** shows that after EuNP-labeled RBD, an obvious protein halo can be observed, indicating that the fluorescent probe (EuNP-RBD) has been successfully prepared. The results show that EuNPs can be successfully conjugated to RBD and that the TRF-BLFIA can distinguish between neutralizing antibodies of other viruses and those produced by COVID-19 vaccine recipients.

### 3.3 Optimization of experimental methods for the TRF-BLFIA

The following parameters were optimized (1): the combination of RBD antigen and ACE2 protein. In order to effectively distinguish antigens purchased from different companies and different models of the same company, we designated four RBDs as RBD-Ag1, RBD-Ag2, RBD-Ag3, and RBD-Ag4, and labeled six ACE2 as ACE2-RP1, ACE2-RP2, ACE2-RP3, ACE2-RP4, ACE2-RP5, and ACE2-RP6 (2); the amount of labeling and coating antigen (3); the signal spray volume (4); the ratio of the signal probe of EuNPs-RBD and EuNPs-goat anti-chicken IgY (5); the sample pad treatment solution (6); the sample diluent (7); the serum dilution factor; and (8) the sample reaction time.

The following experimental conditions were found to give the best results (1): best combination of RBD antigen and ACE2 protein, RBD-Ag3 and ACE2-RP2 (2); optimal amount of labeling and coating antigen, 15 µg RBD-Ag3 and 0.5 µg cm^−1^ ACE2-RP2 (3); optimal signal spray volume, 4 μL cm^−1^ (4); optimal ratio of signal probe of EuNPs-RBD and EuNPs-goat anti-chicken IgY, 11 and 1 (5); optimal sample pad treatment solution, Tris–HCl pH 8.0 (6); optimal sample diluent, PBS containing 0.5% Tween20 (7); serum dilution factor, fivefold dilution; and (8) sample reaction time, 20 min. All condition screening takes the maximum negative and positive difference as the optimal condition, except that the sample reaction time is based on the T-line fluorescence signal difference of negative and positive samples (**Supplementary Figure S1**). Therefore, the optimal conditions were selected as the optimal preparation and reaction conditions for the immunoassay system.

### 3.4 Performance of the TRF-BLFIA

#### 3.4.1 The calibration curve of TRF-BLFIA

**Figure 3:**
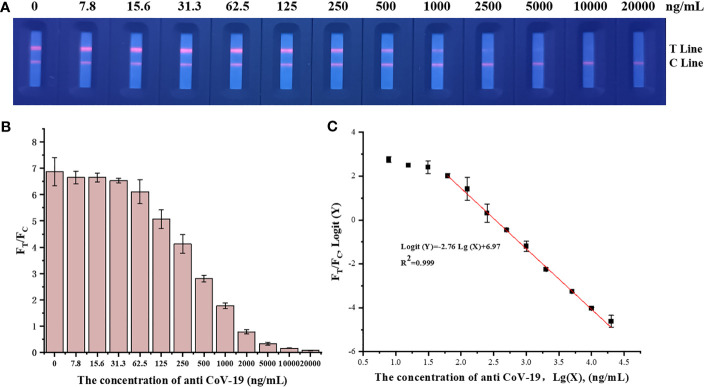
**(A)** A series of concentrations of anti-CoV-19 IgG fluorescence photographs from left to right are 0 ng mL^−1^, 7.8 ng mL^−1^, 15.6 ng mL^−1^, 31.3 ng mL^−1^, 62.5 ng mL^−1^, 125 ng mL^−1^, 250 ng mL^−1^, 500 ng mL^−1^, 1,000 ng mL^−1^, 2,000 ng mL^−1^, 5,000 ng mL^−1^, 10,000 ng mL^−1^ and 20,000 ng mL^−1^. **(B)** Standard curve in which the abscissa is the concentration of anti-CoV-19 IgG, and the ordinate is F_T_/F_C_. **(C)** Standard curve in which the abscissa is the logarithm of the anti-CoV-19 IgG concentration, and the ordinate is the F_T_/F_C_ logarithm.

Fluorescence immunochromatographic strips were tested using anti-CoV-19 IgG standards, and the results are shown in **Figure 3**. The fluorescence immunoassay strip has a linear detection range of 31.3–10,000 ng mL^−1^, Logit(Y)=−2.76Lg(X)+6.97, R^2 = ^0.999.

#### 3.4.2 Determination of TRF-BLFIA specificity

**Figure 4:**
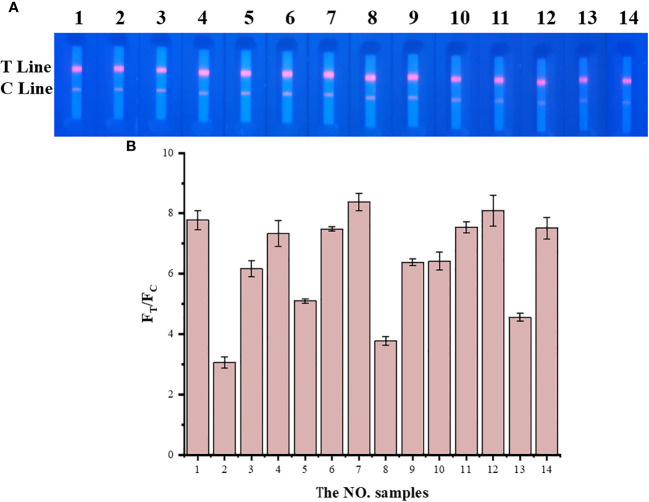
The result of the specificity experiment for multiple pathogens. **(A)** Multiple pathogens assay fluorescence photographs. **(B)** The fluorescence blocking test strip has a significant cross-reaction with samples 5, 8, and 13 and slight cross-reaction with samples 3, 9, and 10. The NO. samples 1–10 correspond, respectively, to negative serum, positive serum, *Staphylococcus aureus*, *Staphylococcus aureus, Staphylococcus haemolyticus*, *Escherichia coli*, *Escherichia coli*, *Enterococcus faecium*, *Candida albicans*, *Klebsiella pneumoniae*, *Klebsiella pneumoniae*, *Klebsiella pneumoniae*, *Stenotrophomonas maltophilia*, and *Aeromonas hydrophila*, respectively.

TRF-BLFIA was tested for specificity using serum from patients infected with multiple common pathogens (such as *S. aureus*, *S. haemolyticus*, *E. coli*, *E. faecium*, *C. albicans*, *K. pneumoniae*, *S. maltophilia*, and *A. hydrophila*) compared with the negative control serum and the positive quality control serum (**Table 1**). As shown in **Figure 4**, the fluorescence blocking test strip has a significant cross-reaction with samples 5, 8, and 13 and slight cross-reacts with samples 3, 9, and 10. A study has reported that the recovered patients with COVID-19 still maintain a strong neutralizing antibody response, including antibodies that can neutralize other coronaviruses in some cases (Zhang et al., 2021b). Based on the above research, we suspect that there may be cross-reaction between some bacteria and SARS-CoV-2 S protein, and we will continue to conduct in-depth research in this area in the future. Meanwhile, to accurately detect clinical samples, this test strip needs to be further improved in specificity.

**Table 1:** Specific experimental pathogen species.

The NO. sample	Pathogens
1	Negative serum
2	Positive serum
3	*Staphylococcus aureus*
4	*Staphylococcus aureus*
5	*Staphylococcus haemolyticus*
6	*Escherichia coli*
7	*Escherichia coli*
8	*Enterococcus faecium*
9	*Candida albicans*
10	*Klebsiella pneumoniae*
11	*Klebsiella pneumoniae*
12	*Klebsiella pneumoniae*
13	*Stenotrophomonas maltophilia*
14	*Aeromonas hydrophila*

#### 3.4.3 Determination of TRF-BLFIA accuracy

The accuracy test results of TRF-BLFIA are shown in **Supplementary Table S1**. Anti-CoV-19 IgG concentrations of 62.5 ng mL^−1^, 250 ng mL^−1^, 500 ng mL^−1^, 1,000 ng mL^−1^, and 5,000 ng mL^−1^ were detected, and the recovery rates were 99%, 104%, 98%, 96%, and 102%, respectively, which met the requirements of 85%–115%.

#### 3.4.4 Determination of TRF-BLFIA repeatability

Samples with concentrations of 250 ng mL^−1^, 500 ng mL^−1^, and 1,000 ng mL^−1^ were added to the same batch of test strips in turn, and each sample was repeated five times. Samples with concentrations of 250 ng mL^−1^, 500 ng mL^−1^, and 1,000 ng mL^−1^ were added, and five batches of test strips were repeated. The average value, standard deviation, and coefficient of variation were calculated, and the intra- and interbatch repeatability of the test strip was evaluated.

The same batch and different batches of TRF-BLFIA strips were used to test the samples of different concentrations, and the results are shown in **Supplementary Tables S2, S3**, CV <10%. The results show that the intra- and interbatch repeatability is acceptable.

#### 3.4.5 Determination of TRF-BLFIA stability

**Figure 5:**
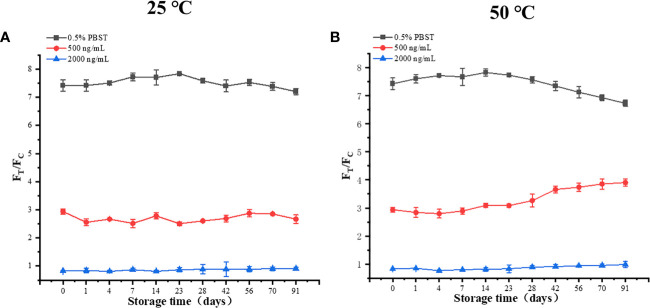
**(A)** The results of three different concentrations after 91 days at 25 °C. **(B)** The results of three different concentrations after 91 days at 50°C.

The stability results of TRF-BLFIA are shown in **Figure 5**. The results of storage at 25°C and 50°C for 91 days show that the values of the three concentrations T/C do not intersect and change stably, which proves that the stability of TRF-BLFIA is acceptable.

### 3.5 Clinical sample analysis of the TRF-BLFIA

**Figure 6:**
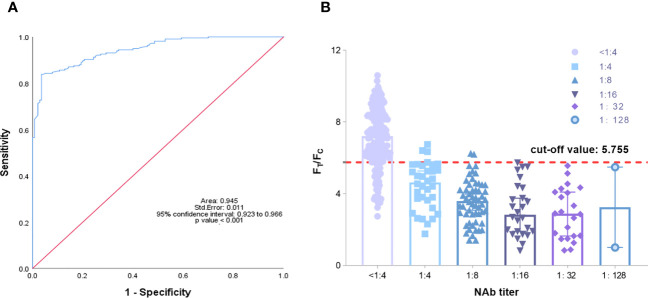
**(A)** Analysis of the ROC curve results of 356 reference serum samples. The area under the curve (AUC) was 0.945, demonstrating that the TRF-BLFIA had significantly high diagnostic value (p < 0.001). **(B)** The neutralization antibody titer was used as the abscissa, and F_T_/F_C_ was used as the ordinate. The graph was drawn by GraphPad Prism 9.0.

Fluorescence immunochromatography and a 2019-nCoV IgG antibody diagnostic kit were used to detect 356 serum samples, including 216 negative samples and 140 positive samples that had been tested by cVNT. An antibody titer ≥1:4 was defined as positive (Marklund et al., 2020). The consistency between the two methods and cVNT was analyzed by SPSS 26.0 software. **Figure 6A** shows that the area under the curve (AUC) of the TRF-BLFIA was 0.945 (95% CI, 0.923–0.966; p<0.0001), indicating that this method has high diagnostic value. The Youden index was calculated according to the sensitivity and specificity of the method, and the F_T_/F_C_ corresponding to the maximum Youden index was taken as the cutoff value of this method. From **Supplementary Table S4**, the cutoff value of this method is determined to be 5.755. Among the 140 positive sera detected by cVNT, 135 positive sera were detected by this method, and the positive coincidence rate reached 96.43%. Among the 216 negative serum samples detected by cVNT, 181 negative serum samples were detected by fluorescence immunochromatography, the negative coincidence rate reached 83.80%, and the total coincidence rate reached 88.76% (**Figure 6B**; **Table 2**). The Kappa coefficient reached 0.773 (p < 0.001), indicating a high degree of consistency between the two methods (**Supplementary Table S5**).

**Table 2:** The coincidence rate between the TRF-BLFIA and cVNT.

Detection method		cVNT		Total
		Negative	Positive	
TRF-BLFIA	Negative	181	5	186
	Positive	35	135	170
	Total	216	140	356
Coincidence rate		83.80%	96.43%	88.76%

It should be noted that at present, the kits for testing SARS-CoV-2 neutralizing antibodies after COVID-19 vaccination has not yet been approved. Most hospitals and medical institutions provide SARS-CoV-2 S-IgG testing. Therefore, we also tested SARS-CoV-2 S-IgG in the samples. Among the samples tested by the 2019-nCoV IgG antibody diagnostic kit, the positive coincidence rate reached 100%, but the negative coincidence rate was only 62.04%, and the total coincidence rate was only 76.97% (**Table 3**). The Kappa coefficient was only 0.562 (p < 0.001) (**Supplementary Table S6**). Although the positive coincidence rate of TRF-BLFIA is slightly lower than that of S-IgG, the negative coincidence rate and the total coincidence rate of TRF-BLFIA are higher than those of S-IgG. Meanwhile, it is reported that there is a contradictory relationship between anti-RBD antibody level and NAbs. Billon-Denis et al. (2021). studied two patients—one who presented with a strong S-IgG immune response that correlated with a low NAb titer, whereas the other had strong S-IgG immune response but high NAb titers. Hence, it is indicated that the detection results of TRF-BLFIA are more accurate and reliable in the evaluation of neutralizing antibodies than S-IgG.

**Table 3:** The coincidence rate between S-IgG and cVNT.

Detection method		cVNT		Total
		Negative	Positive	
S-IgG	Negative	134	0	134
	Positive	82	140	222
	Total	216	140	356
coincidence rate		62.04%	100.00%	76.97%

## 4 Conclusion

In this study, a new neutralizing antibody detection method called TRF-BLFIA was developed using the RBD protein expressed by this research group and the ACE2 protein. This method is sensitive, selective, rapid, and low cost in detecting the neutralizing antibody content in the serum samples of COVID-19 vaccine recipients, and the assay detection results are more reliable and accurate than S-IgG in evaluating the immune efficacy of the vaccine. This method will play an important role in the evaluation of the immune protection of persons previously infected with COVID-19, the neutralizing antibody persistence after vaccination, the evaluation of the time of revaccination, and the effectiveness of neutralizing antibody drugs against COVID-19 in the future. In future studies, to better simulate the process of binding of SARS-CoV-2 to the ACE2 receptor in the body, our research team will continue to design and express the trimeric S protein to further optimize the immune analysis system to more accurately meet the post-pandemic era COVID-19 demand for neutralizing antibody testing brought about by large-scale medical treatment.

## Data availability statement

The raw data supporting the conclusions of this article will be made available by the authors, without undue reservation.

## Ethics statement

The studies involving human participants were reviewed and approved by KY-2020128. The patients/participants provided their written informed consent to participate in this study.

## Author contributions

YL, JH, and YZ contributed equally to this work. YL: conceptualization, methodology, investigation, and writing original draft. JH: formal analysis and investigation. YZ: conceptualization, validation, and data curation. DL: resources. JZ: investigation and validation. RJ: data curation. YW: data curation. ZS: data curation. NX: funding acquisition. CK: supervision and funding acquisition. YT: supervision, funding acquisition, and writing—review and editing. JX: conceptualization, formal analysis, supervision, funding acquisition, and writing—review and editing. All authors have read and agreed to the published version of the manuscript.
