# Supplementary material for: The instantly blocking-based fluorescent immunochromatographic assay for the detection of SARS-CoV-2 neutralizing antibody
Source: Front Cell Infect Microbiol. 2023 Sep 5;13:1203625. doi: 10.3389/fcimb.2023.1203625 (PMC10509472; doi:10.3389/fcimb.2023.1203625)
Supplement: Supplementary file 1 [file DataSheet_1.docx]

**The instantly blocking-based fluorescent immunochromatographic assay for the detection of SARS-CoV-2 neutralizing antibody**

Supplementary data


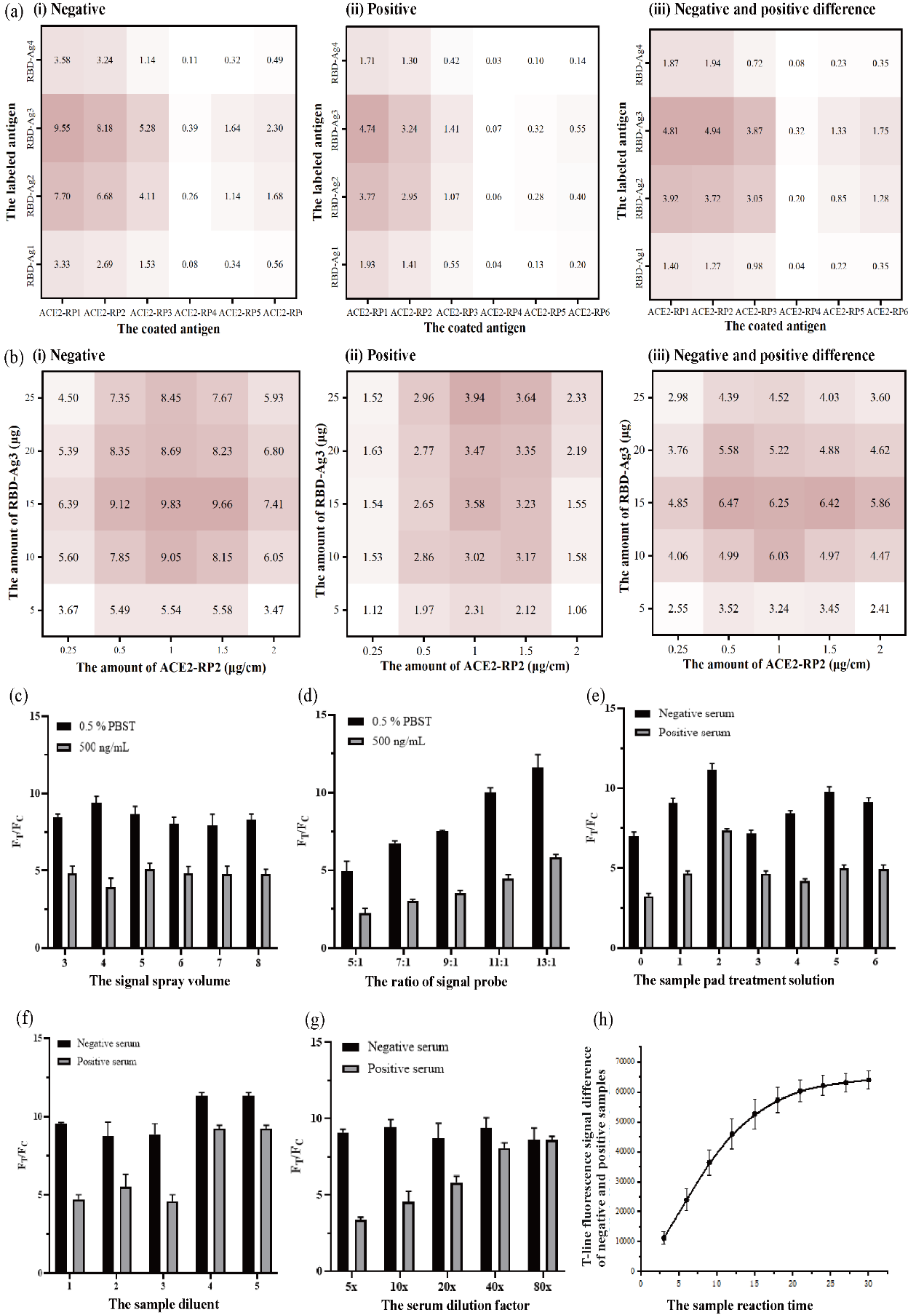


Figure. S1. (a) The maximum difference between negative and positive samples is 4.94, the best coating antigen is ACE2-RP2, and the best labeling antigen is RBD-Ag3. (b) The maximum difference between negative and positive samples is 6.47, optimal amount of labeling and coating antigen: 15ug RBD-Ag3 and 0.5 ug /cm ACE2-RP2 (c) The signal spray volume is 4μL/cm, and the difference between negative and positive samples is the largest, which is the optimal condition. (d) The ratio of signal probe is 11:1, and the difference between negative and positive samples is the largest, which is the optimal condition. (e) The sample pad treatment solution is Tris-HCl (0.1M pH 8.0), and the difference between negative and positive samples is the largest, which is the optimal condition. (f) The sample diluent is 0.5% PBST, and the difference between negative and positive samples is the largest, which is the optimal condition. (g) The serum dilution factor is 5 times, and the difference between negative and positive samples is the largest, which is the optimal condition. (h). The sample reaction time was 20min, and the difference of T-line fluorescence signal between negative and positive samples reached the plateau stage.

Table S1. The result of TRF-BLFIA accuracy

| Samples | 1 | 2 | 3 | 4 | 5 | M(F_T_/F_C_) | SD(F_T_/F_C_) | Recovery(F_T_/F_C_) |
| --- | --- | --- | --- | --- | --- | --- | --- | --- |
| 62.5 | 5.80 | 5.79 | 5.74 | 5.88 | 5.92 | 5.82 | 0.06 | 99% |
| 250 | 3.36 | 3.97 | 3.86 | 3.58 | 4.16 | 3.79 | 0.28 | 104% |
| 500 | 2.67 | 2.61 | 2.49 | 2.56 | 2.48 | 2.56 | 0.07 | 98% |
| 1000 | 1.58 | 1.32 | 1.45 | 1.62 | 1.30 | 1.45 | 0.13 | 96% |
| 5000 | 0.23 | 0.23 | 0.26 | 0.26 | 0.23 | 0.24 | 0.01 | 102% |

Table S2. The results of intrabatch repeatability

| Sample  (ng/mL) | intrabatch values | | | | | M(F_T_/F_C_) | SD(F_T_/F_C_) | CV(F_T_/F_C_) |
| --- | --- | --- | --- | --- | --- | --- | --- | --- |
|  | 1 | 2 | 3 | 4 | 5 |  |  |  |
| 250 | 3.79 | 3.65 | 3.97 | 3.57 | 3.48 | 3.69 | 0.17 | 5% |
| 500 | 2.64 | 2.85 | 2.66 | 2.53 | 2.66 | 2.67 | 0.10 | 4% |
| 1000 | 1.24 | 1.30 | 1.26 | 1.38 | 1.21 | 1.28 | 0.06 | 5% |

Table S3. The results of interbatch repeatability

| Sample  (ng/mL) | interbatch values | | | | | M(F_T_/F_C_) | SD(F_T_/F_C_) | CV(F_T_/F_C_) |
| --- | --- | --- | --- | --- | --- | --- | --- | --- |
|  | 1 | 2 | 3 | 4 | 5 |  |  |  |
| 250 | 3.69 | 3.56 | 3.46 | 3.74 | 3.82 | 3.65 | 0.13 | 4% |
| 500 | 2.67 | 2.51 | 2.86 | 2.53 | 2.72 | 2.66 | 0.13 | 5% |
| 1000 | 1.28 | 1.45 | 1.18 | 1.46 | 1.32 | 1.34 | 0.11 | 8% |

Table S4. Coordinate points on the ROC curve. The corresponding coordinate point when the Youden’s index is the largest is the cut-off value of the fluorescence Immunochromatography.

| Coordinates of the curve | | | | | | | Coordinates of the curve | | | | | | | | |  |
| --- | --- | --- | --- | --- | --- | --- | --- | --- | --- | --- | --- | --- | --- | --- | --- | --- |
| Cut-off value | Sensitivity | | 1-Specificity | | | Youden’s index | | | Cut-off value | Sensitivity | | 1-Specificity | | | Youden’s index | |
| -0.18 | | 1 | | 1 | 0 | | | 5.875 | | | 0.819 | | 0.036 | 0.783 | |  |
| 0.83 | | 1 | | 0.993 | 0.007 | | | 5.885 | | | 0.815 | | 0.036 | 0.779 | |  |
| 0.86 | | 1 | | 0.986 | 0.014 | | | 5.895 | | | 0.81 | | 0.036 | 0.774 | |  |
| 0.94 | | 1 | | 0.979 | 0.021 | | | 5.91 | | | 0.801 | | 0.036 | 0.765 | |  |
| 1.095 | | 1 | | 0.971 | 0.029 | | | 5.925 | | | 0.792 | | 0.036 | 0.756 | |  |
| 1.225 | | 1 | | 0.964 | 0.036 | | | 5.955 | | | 0.787 | | 0.036 | 0.751 | |  |
| 1.33 | | 1 | | 0.957 | 0.043 | | | 5.985 | | | 0.778 | | 0.036 | 0.742 | |  |
| 1.435 | | 1 | | 0.943 | 0.057 | | | 6.015 | | | 0.773 | | 0.036 | 0.737 | |  |
| 1.475 | | 1 | | 0.936 | 0.064 | | | 6.055 | | | 0.769 | | 0.036 | 0.733 | |  |
| 1.485 | | 1 | | 0.929 | 0.071 | | | 6.075 | | | 0.759 | | 0.036 | 0.723 | |  |
| 1.565 | | 1 | | 0.921 | 0.079 | | | 6.095 | | | 0.755 | | 0.036 | 0.719 | |  |
| 1.65 | | 1 | | 0.914 | 0.086 | | | 6.125 | | | 0.745 | | 0.036 | 0.709 | |  |
| 1.665 | | 1 | | 0.9 | 0.1 | | | 6.15 | | | 0.741 | | 0.036 | 0.705 | |  |
| 1.7 | | 1 | | 0.893 | 0.107 | | | 6.17 | | | 0.736 | | 0.036 | 0.7 | |  |
| 1.745 | | 1 | | 0.879 | 0.121 | | | 6.185 | | | 0.731 | | 0.036 | 0.695 | |  |
| 1.775 | | 1 | | 0.871 | 0.129 | | | 6.195 | | | 0.727 | | 0.036 | 0.691 | |  |
| 1.795 | | 1 | | 0.864 | 0.136 | | | 6.215 | | | 0.727 | | 0.029 | 0.698 | |  |
| 1.845 | | 1 | | 0.857 | 0.143 | | | 6.235 | | | 0.722 | | 0.029 | 0.693 | |  |
| 1.9 | | 1 | | 0.85 | 0.15 | | | 6.245 | | | 0.718 | | 0.029 | 0.689 | |  |
| 1.93 | | 1 | | 0.843 | 0.157 | | | 6.255 | | | 0.713 | | 0.021 | 0.692 | |  |
| 1.955 | | 1 | | 0.836 | 0.164 | | | 6.265 | | | 0.704 | | 0.021 | 0.683 | |  |
| 1.97 | | 1 | | 0.829 | 0.171 | | | 6.275 | | | 0.694 | | 0.021 | 0.673 | |  |
| 1.985 | | 1 | | 0.821 | 0.179 | | | 6.285 | | | 0.69 | | 0.021 | 0.669 | |  |
| 2 | | 1 | | 0.814 | 0.186 | | | 6.3 | | | 0.685 | | 0.021 | 0.664 | |  |
| 2.05 | | 1 | | 0.807 | 0.193 | | | 6.315 | | | 0.681 | | 0.021 | 0.66 | |  |
| 2.11 | | 1 | | 0.8 | 0.2 | | | 6.325 | | | 0.671 | | 0.021 | 0.65 | |  |
| 2.175 | | 1 | | 0.793 | 0.207 | | | 6.335 | | | 0.667 | | 0.021 | 0.646 | |  |
| 2.225 | | 1 | | 0.786 | 0.214 | | | 6.345 | | | 0.662 | | 0.021 | 0.641 | |  |
| 2.27 | | 1 | | 0.779 | 0.221 | | | 6.38 | | | 0.653 | | 0.014 | 0.639 | |  |
| 2.33 | | 1 | | 0.771 | 0.229 | | | 6.415 | | | 0.644 | | 0.007 | 0.637 | |  |
| 2.36 | | 1 | | 0.764 | 0.236 | | | 6.435 | | | 0.634 | | 0.007 | 0.627 | |  |
| 2.395 | | 1 | | 0.757 | 0.243 | | | 6.455 | | | 0.63 | | 0.007 | 0.623 | |  |
| 2.46 | | 1 | | 0.75 | 0.25 | | | 6.475 | | | 0.62 | | 0.007 | 0.613 | |  |
| 2.54 | | 1 | | 0.743 | 0.257 | | | 6.525 | | | 0.616 | | 0.007 | 0.609 | |  |
| 2.585 | | 1 | | 0.736 | 0.264 | | | 6.565 | | | 0.611 | | 0.007 | 0.604 | |  |
| 2.61 | | 1 | | 0.729 | 0.271 | | | 6.575 | | | 0.602 | | 0.007 | 0.595 | |  |
| 2.64 | | 1 | | 0.721 | 0.279 | | | 6.605 | | | 0.597 | | 0.007 | 0.59 | |  |
| 2.67 | | 1 | | 0.707 | 0.293 | | | 6.645 | | | 0.593 | | 0.007 | 0.586 | |  |
| 2.715 | | 1 | | 0.7 | 0.3 | | | 6.665 | | | 0.588 | | 0.007 | 0.581 | |  |
| 2.8 | | 0.995 | | 0.7 | 0.295 | | | 6.675 | | | 0.583 | | 0.007 | 0.576 | |  |
| 2.865 | | 0.995 | | 0.679 | 0.316 | | | 6.685 | | | 0.579 | | 0.007 | 0.572 | |  |
| 2.875 | | 0.995 | | 0.671 | 0.324 | | | 6.705 | | | 0.574 | | 0.007 | 0.567 | |  |
| 2.89 | | 0.995 | | 0.664 | 0.331 | | | 6.735 | | | 0.565 | | 0.007 | 0.558 | |  |
| 2.905 | | 0.995 | | 0.657 | 0.338 | | | 6.755 | | | 0.565 | | 0 | 0.565 | |  |
| 2.925 | | 0.995 | | 0.65 | 0.345 | | | 6.765 | | | 0.556 | | 0 | 0.556 | |  |
| 2.95 | | 0.995 | | 0.636 | 0.359 | | | 6.79 | | | 0.551 | | 0 | 0.551 | |  |
| 2.975 | | 0.995 | | 0.629 | 0.366 | | | 6.825 | | | 0.546 | | 0 | 0.546 | |  |
| 3.02 | | 0.995 | | 0.614 | 0.381 | | | 6.85 | | | 0.542 | | 0 | 0.542 | |  |
| 3.065 | | 0.995 | | 0.607 | 0.388 | | | 6.87 | | | 0.537 | | 0 | 0.537 | |  |
| 3.14 | | 0.995 | | 0.6 | 0.395 | | | 6.89 | | | 0.532 | | 0 | 0.532 | |  |
| 3.215 | | 0.995 | | 0.593 | 0.402 | | | 6.985 | | | 0.528 | | 0 | 0.528 | |  |
| 3.24 | | 0.991 | | 0.593 | 0.398 | | | 7.08 | | | 0.523 | | 0 | 0.523 | |  |
| 3.26 | | 0.991 | | 0.579 | 0.412 | | | 7.095 | | | 0.519 | | 0 | 0.519 | |  |
| 3.28 | | 0.991 | | 0.571 | 0.42 | | | 7.105 | | | 0.514 | | 0 | 0.514 | |  |
| 3.3 | | 0.991 | | 0.564 | 0.427 | | | 7.125 | | | 0.509 | | 0 | 0.509 | |  |
| 3.32 | | 0.991 | | 0.557 | 0.434 | | | 7.18 | | | 0.505 | | 0 | 0.505 | |  |
| 3.335 | | 0.991 | | 0.55 | 0.441 | | | 7.23 | | | 0.495 | | 0 | 0.495 | |  |
| 3.375 | | 0.991 | | 0.543 | 0.448 | | | 7.255 | | | 0.491 | | 0 | 0.491 | |  |
| 3.42 | | 0.991 | | 0.536 | 0.455 | | | 7.275 | | | 0.486 | | 0 | 0.486 | |  |
| 3.465 | | 0.991 | | 0.529 | 0.462 | | | 7.29 | | | 0.477 | | 0 | 0.477 | |  |
| 3.505 | | 0.986 | | 0.529 | 0.457 | | | 7.305 | | | 0.472 | | 0 | 0.472 | |  |
| 3.525 | | 0.981 | | 0.529 | 0.452 | | | 7.315 | | | 0.468 | | 0 | 0.468 | |  |
| 3.545 | | 0.981 | | 0.521 | 0.46 | | | 7.33 | | | 0.463 | | 0 | 0.463 | |  |
| 3.59 | | 0.981 | | 0.514 | 0.467 | | | 7.345 | | | 0.454 | | 0 | 0.454 | |  |
| 3.635 | | 0.981 | | 0.507 | 0.474 | | | 7.355 | | | 0.444 | | 0 | 0.444 | |  |
| 3.655 | | 0.981 | | 0.5 | 0.481 | | | 7.385 | | | 0.44 | | 0 | 0.44 | |  |
| 3.68 | | 0.981 | | 0.486 | 0.495 | | | 7.415 | | | 0.431 | | 0 | 0.431 | |  |
| 3.695 | | 0.977 | | 0.486 | 0.491 | | | 7.43 | | | 0.426 | | 0 | 0.426 | |  |
| 3.715 | | 0.972 | | 0.486 | 0.486 | | | 7.445 | | | 0.417 | | 0 | 0.417 | |  |
| 3.745 | | 0.972 | | 0.471 | 0.501 | | | 7.455 | | | 0.412 | | 0 | 0.412 | |  |
| 3.765 | | 0.968 | | 0.464 | 0.504 | | | 7.465 | | | 0.407 | | 0 | 0.407 | |  |
| 3.775 | | 0.963 | | 0.464 | 0.499 | | | 7.475 | | | 0.403 | | 0 | 0.403 | |  |
| 3.785 | | 0.963 | | 0.457 | 0.506 | | | 7.485 | | | 0.398 | | 0 | 0.398 | |  |
| 3.795 | | 0.958 | | 0.457 | 0.501 | | | 7.5 | | | 0.389 | | 0 | 0.389 | |  |
| 3.805 | | 0.958 | | 0.45 | 0.508 | | | 7.515 | | | 0.384 | | 0 | 0.384 | |  |
| 3.835 | | 0.954 | | 0.429 | 0.525 | | | 7.53 | | | 0.38 | | 0 | 0.38 | |  |
| 3.88 | | 0.949 | | 0.421 | 0.528 | | | 7.565 | | | 0.37 | | 0 | 0.37 | |  |
| 3.94 | | 0.949 | | 0.414 | 0.535 | | | 7.595 | | | 0.361 | | 0 | 0.361 | |  |
| 4.02 | | 0.949 | | 0.407 | 0.542 | | | 7.61 | | | 0.356 | | 0 | 0.356 | |  |
| 4.07 | | 0.949 | | 0.4 | 0.549 | | | 7.64 | | | 0.352 | | 0 | 0.352 | |  |
| 4.09 | | 0.944 | | 0.4 | 0.544 | | | 7.72 | | | 0.347 | | 0 | 0.347 | |  |
| 4.11 | | 0.944 | | 0.386 | 0.558 | | | 7.795 | | | 0.343 | | 0 | 0.343 | |  |
| 4.14 | | 0.944 | | 0.379 | 0.565 | | | 7.86 | | | 0.338 | | 0 | 0.338 | |  |
| 4.175 | | 0.944 | | 0.371 | 0.573 | | | 7.915 | | | 0.333 | | 0 | 0.333 | |  |
| 4.205 | | 0.944 | | 0.364 | 0.58 | | | 7.945 | | | 0.329 | | 0 | 0.329 | |  |
| 4.225 | | 0.944 | | 0.343 | 0.601 | | | 7.98 | | | 0.324 | | 0 | 0.324 | |  |
| 4.235 | | 0.94 | | 0.343 | 0.597 | | | 8.015 | | | 0.319 | | 0 | 0.319 | |  |
| 4.255 | | 0.94 | | 0.336 | 0.604 | | | 8.045 | | | 0.315 | | 0 | 0.315 | |  |
| 4.275 | | 0.935 | | 0.336 | 0.599 | | | 8.095 | | | 0.31 | | 0 | 0.31 | |  |
| 4.3 | | 0.931 | | 0.329 | 0.602 | | | 8.165 | | | 0.301 | | 0 | 0.301 | |  |
| 4.335 | | 0.931 | | 0.321 | 0.61 | | | 8.2 | | | 0.296 | | 0 | 0.296 | |  |
| 4.375 | | 0.931 | | 0.314 | 0.617 | | | 8.215 | | | 0.292 | | 0 | 0.292 | |  |
| 4.405 | | 0.931 | | 0.307 | 0.624 | | | 8.235 | | | 0.282 | | 0 | 0.282 | |  |
| 4.435 | | 0.931 | | 0.293 | 0.638 | | | 8.27 | | | 0.278 | | 0 | 0.278 | |  |
| 4.475 | | 0.926 | | 0.293 | 0.633 | | | 8.3 | | | 0.273 | | 0 | 0.273 | |  |
| 4.495 | | 0.926 | | 0.286 | 0.64 | | | 8.315 | | | 0.259 | | 0 | 0.259 | |  |
| 4.51 | | 0.926 | | 0.279 | 0.647 | | | 8.33 | | | 0.255 | | 0 | 0.255 | |  |
| 4.525 | | 0.926 | | 0.271 | 0.655 | | | 8.365 | | | 0.245 | | 0 | 0.245 | |  |
| 4.54 | | 0.921 | | 0.271 | 0.65 | | | 8.395 | | | 0.236 | | 0 | 0.236 | |  |
| 4.555 | | 0.921 | | 0.264 | 0.657 | | | 8.405 | | | 0.227 | | 0 | 0.227 | |  |
| 4.565 | | 0.921 | | 0.257 | 0.664 | | | 8.425 | | | 0.222 | | 0 | 0.222 | |  |
| 4.58 | | 0.917 | | 0.25 | 0.667 | | | 8.465 | | | 0.218 | | 0 | 0.218 | |  |
| 4.595 | | 0.912 | | 0.25 | 0.662 | | | 8.515 | | | 0.213 | | 0 | 0.213 | |  |
| 4.625 | | 0.912 | | 0.243 | 0.669 | | | 8.545 | | | 0.208 | | 0 | 0.208 | |  |
| 4.66 | | 0.907 | | 0.243 | 0.664 | | | 8.56 | | | 0.204 | | 0 | 0.204 | |  |
| 4.695 | | 0.903 | | 0.243 | 0.66 | | | 8.58 | | | 0.199 | | 0 | 0.199 | |  |
| 4.73 | | 0.903 | | 0.236 | 0.667 | | | 8.6 | | | 0.194 | | 0 | 0.194 | |  |
| 4.785 | | 0.903 | | 0.229 | 0.674 | | | 8.67 | | | 0.181 | | 0 | 0.181 | |  |
| 4.84 | | 0.903 | | 0.221 | 0.682 | | | 8.745 | | | 0.176 | | 0 | 0.176 | |  |
| 4.88 | | 0.903 | | 0.214 | 0.689 | | | 8.775 | | | 0.171 | | 0 | 0.171 | |  |
| 4.925 | | 0.903 | | 0.207 | 0.696 | | | 8.795 | | | 0.167 | | 0 | 0.167 | |  |
| 4.95 | | 0.898 | | 0.207 | 0.691 | | | 8.855 | | | 0.162 | | 0 | 0.162 | |  |
| 4.965 | | 0.898 | | 0.2 | 0.698 | | | 8.915 | | | 0.157 | | 0 | 0.157 | |  |
| 4.98 | | 0.894 | | 0.2 | 0.694 | | | 8.935 | | | 0.153 | | 0 | 0.153 | |  |
| 5.01 | | 0.889 | | 0.193 | 0.696 | | | 8.98 | | | 0.148 | | 0 | 0.148 | |  |
| 5.06 | | 0.884 | | 0.193 | 0.691 | | | 9.015 | | | 0.144 | | 0 | 0.144 | |  |
| 5.1 | | 0.88 | | 0.193 | 0.687 | | | 9.025 | | | 0.139 | | 0 | 0.139 | |  |
| 5.115 | | 0.88 | | 0.186 | 0.694 | | | 9.04 | | | 0.13 | | 0 | 0.13 | |  |
| 5.125 | | 0.875 | | 0.186 | 0.689 | | | 9.06 | | | 0.125 | | 0 | 0.125 | |  |
| 5.14 | | 0.875 | | 0.179 | 0.696 | | | 9.09 | | | 0.12 | | 0 | 0.12 | |  |
| 5.18 | | 0.875 | | 0.171 | 0.704 | | | 9.115 | | | 0.111 | | 0 | 0.111 | |  |
| 5.215 | | 0.875 | | 0.164 | 0.711 | | | 9.125 | | | 0.106 | | 0 | 0.106 | |  |
| 5.235 | | 0.87 | | 0.164 | 0.706 | | | 9.135 | | | 0.102 | | 0 | 0.102 | |  |
| 5.255 | | 0.87 | | 0.157 | 0.713 | | | 9.19 | | | 0.097 | | 0 | 0.097 | |  |
| 5.275 | | 0.87 | | 0.143 | 0.727 | | | 9.25 | | | 0.093 | | 0 | 0.093 | |  |
| 5.295 | | 0.866 | | 0.143 | 0.723 | | | 9.275 | | | 0.088 | | 0 | 0.088 | |  |
| 5.31 | | 0.861 | | 0.136 | 0.725 | | | 9.3 | | | 0.083 | | 0 | 0.083 | |  |
| 5.375 | | 0.861 | | 0.129 | 0.732 | | | 9.315 | | | 0.079 | | 0 | 0.079 | |  |
| 5.455 | | 0.856 | | 0.129 | 0.727 | | | 9.335 | | | 0.074 | | 0 | 0.074 | |  |
| 5.485 | | 0.856 | | 0.121 | 0.735 | | | 9.365 | | | 0.069 | | 0 | 0.069 | |  |
| 5.495 | | 0.856 | | 0.114 | 0.742 | | | 9.39 | | | 0.065 | | 0 | 0.065 | |  |
| 5.51 | | 0.856 | | 0.107 | 0.749 | | | 9.42 | | | 0.06 | | 0 | 0.06 | |  |
| 5.535 | | 0.852 | | 0.107 | 0.745 | | | 9.49 | | | 0.056 | | 0 | 0.056 | |  |
| 5.555 | | 0.852 | | 0.1 | 0.752 | | | 9.55 | | | 0.051 | | 0 | 0.051 | |  |
| 5.585 | | 0.847 | | 0.079 | 0.768 | | | 9.585 | | | 0.046 | | 0 | 0.046 | |  |
| 5.615 | | 0.843 | | 0.079 | 0.764 | | | 9.615 | | | 0.042 | | 0 | 0.042 | |  |
| 5.625 | | 0.843 | | 0.071 | 0.772 | | | 9.645 | | | 0.037 | | 0 | 0.037 | |  |
| 5.635 | | 0.843 | | 0.064 | 0.779 | | | 9.675 | | | 0.032 | | 0 | 0.032 | |  |
| 5.65 | | 0.843 | | 0.057 | 0.786 | | | 9.705 | | | 0.028 | | 0 | 0.028 | |  |
| 5.695 | | 0.843 | | 0.05 | 0.793 | | | 9.76 | | | 0.023 | | 0 | 0.023 | |  |
| 5.74 | | 0.838 | | 0.043 | 0.795 | | | 9.82 | | | 0.019 | | 0 | 0.019 | |  |
| 5.755 | | 0.838 | | 0.036 | 0.802 | | | 9.905 | | | 0.014 | | 0 | 0.014 | |  |
| 5.775 | | 0.833 | | 0.036 | 0.797 | | | 10.125 | | | 0.009 | | 0 | 0.009 | |  |
| 5.795 | | 0.829 | | 0.036 | 0.793 | | | 10.445 | | | 0.005 | | 0 | 0.005 | |  |
| 5.835 | | 0.824 | | 0.036 | 0.788 | | | 11.6 | | | 0 | | 0 | 0 | |  |

Table S5. The symmetric measures between the TRF-BLFIA and cVNT.

|  | Value | Asymp. Std. Error^a^ | Approx. T^b^ | Approx. Sig. |
| --- | --- | --- | --- | --- |
| Measure of Agreement Kappa | 0.773 | 0.033 | 14.803 | 0.000 |
| N of valid Cases | 356 |  |  |  |

a. Not assuming the null hypothesis.

b Using the asymptotic standard error assuming the null hypothesis.

Table S6. The symmetric measures between S-IgG and cVNT.

|  | Value | Asymp. Std. Error^a^ | Approx. T^b^ | Approx. Sig. |
| --- | --- | --- | --- | --- |
| Measure of Agreement Kappa | 0.562 | 0.038 | 11.802 | 0.000 |
| N of valid Cases | 356 |  |  |  |

a. Not assuming the null hypothesis.

b Using the asymptotic standard error assuming the null hypothesis.
